# The contribution of apolipoprotein E genetic variation to dementia risk in British South Asians

**DOI:** 10.1093/braincomms/fcag141

**Published:** 2026-04-17

**Authors:** Benjamin M Jacobs, Avinash Chandra, Isabelle Francesca Foote, Faiza Durrani, Sheena Waters, Yue Liu, Petroula Proitsi, Genes & Health Research Team, Eamonn Maher, Shabana Chaudhary, Joseph Gafton, Karen A Hunt, Shapna Hussain, Kamrul Islam, Mohammed Bodrul Mazid, Elizabeth Owor, Jessry Russell, Nishat Safa, John Solly, Marie Spreckley, David A Van Heel, Jan Whalley, Ishevanhu Zengeya, Emily Mantle, Shaheen Akhtar, Samina Ashraf, Dan Mason, John Wright, Daniel MacArthur, Michael Simpson, Richard C Trembath, Gerome Breen, Raymond Chung, Sang Hyuck Lee, Omar Asgar, Joanne Harvey, Karen Tricker, Caroline Winckley, Hanifa Khatun, Amna Asif, Claudia Langenberg, Grainne Colligan, Ceri Durham, Bill Newman, Ahsan Khan, Hilary Martin, Teng Heng, Matt Hurles, Vivek Iyer, Georgios Kalantzis, Vladimir Ovchinnikov, Iaroslav Popov, Klaudia Walter, Panos Deloukas, David Collier, Ana Angel, Saeed Bidi, Fabiola Eto, Sarah Finer, Chris Griffiths, Sam Hodgson, Benjamin M Jacobs, Rohini Mathur, Caroline Morton, Asma Qureshi, Stuart Rison, Annum Salman, Miriam Samuel, Moneeza K Siddiqui, Daniel Stow, Sabina Yasmin, Julia Zöllner, Sheik Dowlut, Cara L Croft, Dylan M Williams, Sarah Finer, Stuart Rison, Arnab Mandal, Moneeza Siddiqui, David A van Heel, Charles R Marshall

**Affiliations:** Wolfson Institute of Population Health, Queen Mary University of London, London EC1M 6BQ, UK; Department of Neurology, Royal London Hospital, Barts Health NHS Trust, London E1 1FR, UK; Wolfson Institute of Population Health, Queen Mary University of London, London EC1M 6BQ, UK; Wolfson Institute of Population Health, Queen Mary University of London, London EC1M 6BQ, UK; Academic Centre for Health Ageing, Barts Health NHS Trust, London E11 1NR, UK; Wolfson Institute of Population Health, Queen Mary University of London, London EC1M 6BQ, UK; Wolfson Institute of Population Health, Queen Mary University of London, London EC1M 6BQ, UK; Wolfson Institute of Population Health, Queen Mary University of London, London EC1M 6BQ, UK; Wolfson Institute of Population Health, Queen Mary University of London, London EC1M 6BQ, UK; Centre for Neuroscience, Surgery & Trauma, The Blizard Institute, Queen Mary University of London, London E1 2AT, UK; Division of Psychiatry, University College London, London W1T 7NF, UK; Unit for Lifelong Health & Ageing, University College London, London WC1E 6BT, UK; Wolfson Institute of Population Health, Queen Mary University of London, London EC1M 6BQ, UK; Wolfson Institute of Population Health, Queen Mary University of London, London EC1M 6BQ, UK; Wolfson Institute of Population Health, Queen Mary University of London, London EC1M 6BQ, UK; Wolfson Institute of Population Health, Queen Mary University of London, London EC1M 6BQ, UK; Centre for Neuroscience, Surgery & Trauma, The Blizard Institute, Queen Mary University of London, London E1 2AT, UK; Wolfson Institute of Population Health, Queen Mary University of London, London EC1M 6BQ, UK; Department of Neurology, Royal London Hospital, Barts Health NHS Trust, London E1 1FR, UK

**Keywords:** genetics, dementia, APOE, south Asian, ancestry

## Abstract

Understanding the genetic basis of dementia in diverse populations is essential to ensure that efforts to predict, prevent and treat dementia are equitable. The strongest genetic risk factor for dementia—*APOE* genotype—has not been assessed in population-scale cohorts of South Asian ancestry. To test whether *APOE* variation is associated with all-cause dementia in British South Asians, we analysed data from 51 104 volunteers in the Genes & Health study—a cohort study of British Bangladeshi and British Pakistani individuals who have undergone genotyping and consented for lifelong (England & Wales National Health Service) linkage to healthcare records. All-cause dementia was defined using electronic healthcare records. *APOE* genotypes were defined using phased, imputed genotype data. Cox proportional hazards models were used to assess the relationship between *APOE* genotype and dementia. Population attributable fractions were calculated for each *APOE* genotype. Phenome-wide association tests were performed to determine the impact of *APOE* genotype on a range of traits and diseases. We identified 614 cases of dementia and 50 490 controls without any dementia diagnostic codes. Dementia cases (*n* = 614, of which 451 [73.5%] were incident) were recruited at an older age (median 71.3 versus 39.0), were more likely to be male (58.6% versus 44.5%), and were more likely to carry at least one *APOE* ε4 allele (26.4% versus 19.6%) than controls without dementia (*n* = 50 490). The *APOE* ε4 allele was associated with all-cause dementia in a dose-dependent fashion (*APOE* ε4/ε4: Hazard ratio [HR] 2.7, 95% CI 1.7–4.2, *P* < 0.0001; *APOE* ε4/ε3: HR 1.5, 95% CI 1.2–1.8, *P* < 0.001; Cox multivariable regression models adjusted for age at recruitment, gender, genetic principal components 1–10, and genetic ancestry; all models used *APOE* ε3/ε3 as reference). The overall proportion of all-cause dementia cases which could be attributed to this allele was 12.9% (95% CI −5.9%–25.5%). *APOE* ε4 was also associated with elevated triglycerides and low-density lipoprotein (LDL) cholesterol. *APOE* ε4—the major genetic risk factor for sporadic dementia in European-ancestry populations—has a similar impact on dementia risk in British South Asians. Prediction and prevention studies aiming to use *APOE* to identify people at high risk of dementia should be inclusive of all ancestries. Genetic analysis of dementia risk in diverse populations is essential for ensuring that the downstream benefits—for prediction, prevention and drug discovery—are shared equitably.

## Graphical Abstract

**Graphical Abstract:**
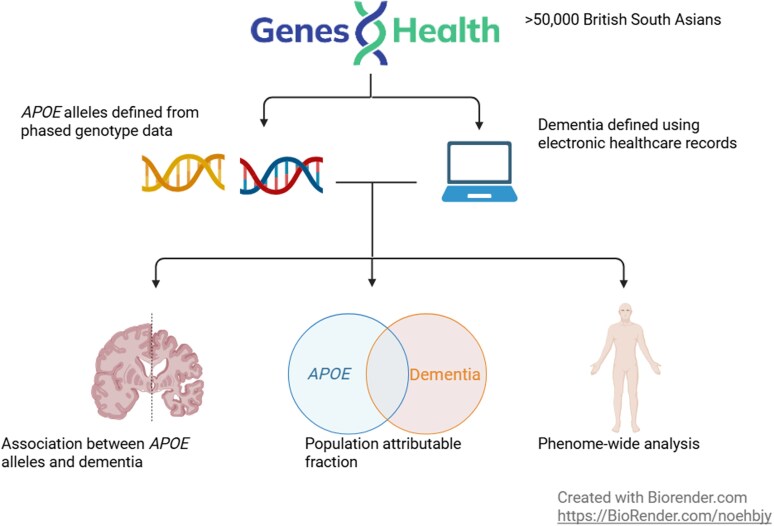

## Teaser

Jacobs *et al.* report that genetic variants in the *apolipoprotein E* gene are associated with all-cause dementia in a cohort of >50 000 British South Asians. They estimate that the *APOE* ε4 allele accounts for 12.9% of dementia in this population, which is lower than estimates from European-ancestry cohorts.

## Introduction

Genome-wide association studies (GWAS) of sporadic Alzheimer’s disease (AD) have demonstrated that AD has a strong genetic component driven by common variation in at least 75 risk loci.^1,2^ Variation at the *APOE* locus (encoding apolipoprotein E; APOE) alone is estimated to account for over one third of cases,^3^ however, this is based almost entirely on evidence from those of European ancestry.

Common coding variants in *APOE* define three common APOE protein isoforms—**ε**2, **ε**3 and **ε**4—of which **ε**4 is associated with increased risk of AD in a dose-dependent fashion and **ε**2 with decreased risk with respect to the most common allele, **ε**3.^4,5^ The association between haplotypes at the *APOE* locus and AD has been consistently demonstrated across cohorts,^1,6,7^ including populations of European, recently admixed (African American), Hispanic and East Asian ancestry. The association between *APOE* genetic variation and dementia risk has not been evaluated at scale in a population of South Asian ancestry, although small studies in Pakistani and Indian populations have suggested an effect similar to that seen in European populations.^8-12^

British South Asians are at higher dementia risk and tend to be diagnosed with dementia at a younger age,^13,14^ experience mortality sooner after a dementia diagnosis,^13^ and have a higher prevalence of dementia risk factors, including deprivation, dyslipidaemia and type 2 diabetes mellitus.^15,16^ Understanding the genetic basis of dementia in this population is essential for prediction and prevention efforts, especially given a surge in forecasted dementia cases in the coming decades.^17^ To date, few studies have examined the prevalence of *APOE* variants and the magnitude of their associations with dementia in a population-scale cohort of South Asian ancestry.

We therefore aimed to consider whether the major European-ancestry genetic risk factor for sporadic dementia—*APOE* genotype—was associated with dementia and other health-related phenotypic outcomes in a cohort of ∼70 000 British South Asian participants in the Genes & Health study. We hypothesized that both allele frequencies and effects on dementia risk might vary relative to European ancestry populations.

## Materials and methods

### Cohort

Genes & Health (G&H) is a longitudinal cohort study of self-identified British Bangladeshi and British Pakistani individuals with genetic data (array genotyping and exome sequencing) and linked electronic healthcare records for over 70 000 healthy volunteers, with recruitment expected to expand to over 100 000 individuals.^16^

### Genetic data & definition of *APOE* haplotypes

G&H volunteers were genotyped from saliva using the Illumina Global Screening Array version 3.0. Genotype quality control procedures have been previously described.^18-20^  *APOE* haplotypes were determined from phased genotype data imputed to the Topmed-r3 reference.^21^ Genotypes at two coding variants—rs429358 and rs7412—define the common *APOE* haplotypes^22^ as follows: rs429358_T_—rs7412_T_ (**ε**2), rs429358_T_—rs7412_C_ (**ε**3), rs429358_C_—rs7412_C_ (**ε**4). We used data from the January 2024 data freeze, comprising imputed genetic data for 51 166 volunteers and excluded 62 volunteers of ambiguous genetic ancestry, giving a final sample size of 51 104.

### Phenotype definitions

All-cause dementia cases were identified using a custom codelist applied to multiple sources of linked electronic healthcare records, including NHS Digital Hospital Episode Statistics, primary care data, and Barts Health and Bradford Teaching Hospitals secondary care records. All-cause dementia was used as the primary outcome as this both maximizes the sample size (and thus statistical power), and because more precise, clinically-subtyped diagnoses of dementia phenotypes are generally unreliable in electronic healthcare records.^23^ In secondary sensitivity analyses, we used Alzheimer’s Dementia (ICD code G30 or F00), unspecified dementia (ICD code F03) and vascular dementia (F01) as the outcome measure. Phenotypes were curated as part of a bespoke Python pipeline. All code and codelists used for phenotype generation are available at https://github.com/genes-and-health/BI_PY.

### Statistical analysis

#### Demographics

Demographic data are presented with medians and interquartile ranges for continuous variables and as numbers and percentages for categorical variables.

#### Allele frequency comparison

We tested for statistical enrichment/depletion of the *APOE*  **ε**4 allele in the Genes & Health cohort using a two-tailed binomial test, comparing the proportion of **ε**4 carriers and non-carriers with the proportion of carriers observed in the subset of ∼340 000 White British UK Biobank volunteers.^24^

#### Association of *APOE* haplotypes with dementia

Survival analysis was conducted using Cox proportional hazards models. As individuals were recruited from the age of 16 or over, the ‘time-to-event’ was defined as the time from the age of 16 to the age of dementia diagnosis. For controls, the censoring time was defined as December 2024 when the last phenotype data extract was performed. Models were adjusted for age, gender, the first ten genetic principal components and binary genetic ancestry (inferred Pakistani or Bangladeshi ancestry). For models, the **ε**3/**ε**3 allele was used as the reference, as this is the most common allele. Each pair of alleles was considered separately (i.e. a ‘genotypic’ model). Hazard ratios are expressed with 95% confidence intervals and *P* values for the null hypothesis that the ratio = 1. Models were inspected for the proportional hazards assumption using Schoenfeld residuals and for linearity. As sensitivity analyses, we repeated the analysis using fewer covariates (age and gender alone), using logistic regression models instead of Cox models (adjusted for identical covariates), we used gender-stratified models, we repeated the analysis using coded ‘Alzheimer’s dementia’ rather than all-cause dementia, and we restricted the analysis to those recruited over the age of 60 and excluded cases diagnosed prior to 60 (note for these models age 60, rather than age 16, was used as time zero for survival analyses).

#### Population attributable fraction (PAF)

We estimated the PAF for all-cause dementia by using the stratified exposure methods described in Hanley (2001)^25^ and used in Williams *et al.* (2025).^3^ To do so, we first used a logistic regression model of the form dementia ∼ *APOE* genotype + covariates, using the low-risk, rare ε2/ε2 genotype as reference, to derive an estimate of the Odds Ratio for dementia given each combination of *APOE* alleles. This means individuals with intermediate risk genotypes (carriers of ε3) are not subsumed into the reference group, which would be expected to bias downwards proportions attributable to risk alleles. We calculated the ‘case fraction’, i.e. the proportion of all cases accounted for by each genotype (i.e. *N* cases/total cases) and estimated the genotype-specific PAF as (OR—1)/(OR × case fraction). The overall PAF attributable to either ε3 or ε4 was estimated by summing the genotype-specific PAFs over all genotypes containing ε3 or ε4 (ε3/ε3, ε3/ε4, ε3/ε2, ε4/ε2, ε4/ε4). To estimate the PAF attributable to ε3 or ε4 alone, we decomposed the relative contributions of ε3 and ε4 to the effect estimate for the ε3/ε4 genotype using the approach described in Williams *et al.* (2025)^3^; specifically, the ratio of the Odds Ratios for ε4/ε2 and ε3/ε2 was used to derive a weight (ratio/ratio + 1 for ε4; 1—this weight for ε3) quantifying the proportion of the PAF for ε4/ε3 attributable to each allele individually. Confidence intervals were calculated by calculating the genotype-specific PAF for each stratum using the lower or upper bound of the effect estimate (OR +/− 1.96×standard error) and summing these PAFs together. The confidence estimates for the decomposed effect of ε3/ε4 were derived using the upper and lower bounds of the ratio.

#### Phenome-wide association study

We analysed the association of *APOE* alleles relative to ε3/ε3 with all available binary (disease) traits with at least 500 cases and all quantitative traits with *N* > 1000. We used logistic (for binary traits) and linear (for quantitative) traits adjusted for the same covariates as in the primary Cox models (age, gender, first ten PCs and ancestry). In the text, we report associations surpassing a study-wide Bonferroni threshold correction to maintain an alpha of 5% (i.e. *P* = 0.0002 = 0.05/236 tests).

## Results

### *APOE* ε4 prevalence in British south asians

We analysed genetic and healthcare data from 51 104 British South Asian participants in the Genes & Health study (**methods**). Demographic characteristics of identified dementia cases and controls are shown in the table (Table 1). Both variants used to define *APOE* alleles were common and well-imputed in G&H: rs429358 (chr19:44908684:T:C, Minor allele frequency [MAF] = 0.10, Imputation quality score [INFO] = 0.95); rs7412 (chr19:44908822:C:T, MAF = 0.04, INFO = 0.95). For both of these *APOE* variants, the allele frequency was consistent with estimates from reference populations of South Asian (SAS) ancestry but lower than in Non-Finnish European (NFE) reference samples (rs429358: MAF_G&H_ = 10%, MAF_gnomAD-SAS_ = 10.1%,^26^ MAF_gnomAD-NFE_ = 15.1%; rs7412 : MAF_G&H_ = 4%, MAF_gnomAD_ = 4.2%, MAF_gnomAD-NFE_ = 7.8%). The overall allele frequencies for *APOE* haplotypes in G&H were 10.4% for *APOE* ε4, 85.1% for *APOE* ε3 and 4.4% for *APOE* ε2. The allele frequency of *APOE* ε4 was 11.3% in the participants of inferred Bangladeshi ancestry and 9.2% in participants of inferred Pakistani ancestry.

**Table 1:** Demographic characteristics

	Dementia	Controls
*N*	614	50 490
Age at recruitment (median, [IQR])	71.3 [15.9]	39.0 [17.5]
Gender (*n*, %)		
Female	254 [41.4%]	28 018 [55.5%]
Male	360 [58.6%]	22 472 [44.5%]
APOE genotype		
ε2/ε2	<10 [<1.7%]	145 [0.3%]
ε2/ε3	39 [6.4%]	3707 [7.3%]
ε2/ε4	<10 [<1.7%]	472 [0.9%]
ε3/ε3	412 [67.1%]	36 738 [72.8%]
ε3/ε4	133 [21.7%]	8831 [17.5%]
ε4/ε4	20 [3.3%]	597 [1.2%]
APOE ε4 alleles		
0	452 [73.6%]	40 590 [80.4%]
1	142 [23.1%]	9303 [18.4%]
2 (ε4/ε4)	20 [3.3%]	597 [1.2%]
APOE allele frequency		
ε2	4.1%	4.4%
ε3	81.1%	85.2%
ε4	14.8%	10.4%
Inferred genetic ancestry		
Bangladeshi	335 [54.6%]	29 614 [58.7%]
Pakistani	279 [45.4%]	20 876 [41.3%]
Age at diagnostic code (median, IQR)	73.3 [16.0]	

Categorical variables are present as *N* (count) and percentage (in square brackets). Continuous variables are presented as median and interquartile range (in square brackets). Note that for the allele frequencies, the *N* is not shown—the denominator is the total number of alleles (i.e. *N* * 2).

Dementia cases (*n* = 614, of which 451 [73.5%] were incident) were recruited at an older age (median 71.3 versus 39.0), were more likely to be male (58.6% versus 44.5%), and were more likely to carry at least one *APOE* ε4 allele (26.4% versus 19.6%) than controls without dementia (*n* = 50 490). There are currently limited data on dementia phenotypes within the Genes & Health cohort. Of the 614 dementia cases, 77 (12.5%) had a coded diagnosis of AD, 125 (20.4%) had a coded diagnosis of vascular dementia, and 210 (34.2%) had a coded diagnosis of unspecified dementia.

**Figure 1:**
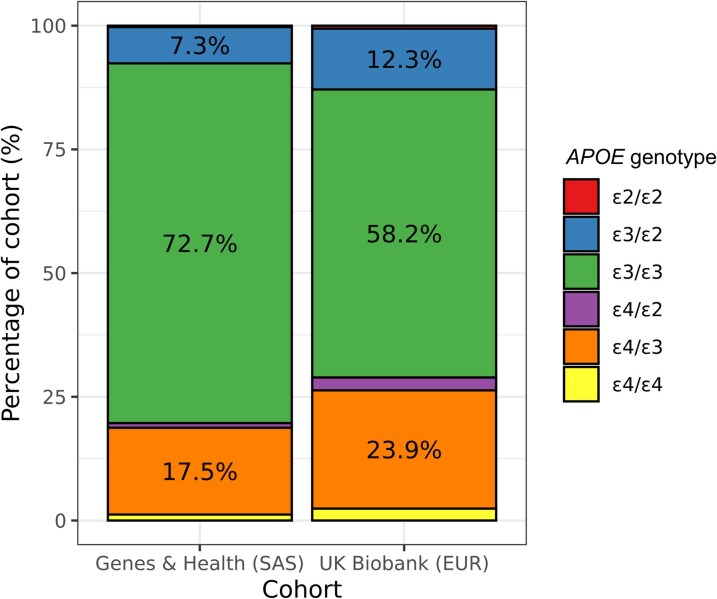
**Frequency of APOE genotypes in British south asians.** Stacked barplots show the distribution of *APOE* genotypes (proportions) for the Genes & Health cohort (left), comprising British Bangladeshi and British Pakistani volunteers, compared with UK Biobank participants of European ancestry (right). The components of the barplot are coloured according to inferred *APOE* genotype. Percentage labels are shown for percentages > 5%. SAS—South Asian ancestry. EUR—European ancestry.

Overall, 19.7% of the population carried at least one *APOE* ε4 allele (18.5% heterozygous, 1.2% homozygous) and 97.6% carried at least one *APOE* ε3 allele (24.9% heterozygous, 72.7% homozygous). The proportion of British South Asians who carry at least one *APOE* ε4 allele is therefore somewhat lower than White British people (Fig. 1; 19.7% versus 28.8% in UK Biobank,^24^ two-tailed Binomial test *P* < 2 × 10^−16^), but consistent with prior estimates from cohorts of South Asian ancestry (e.g. 19.4% in an Indian population^8^ and 18.4% among the few self-identified British South Asians in UK Biobank^27^).

### Association of *APOE* alleles with dementia in genes & health

**Figure 2:**
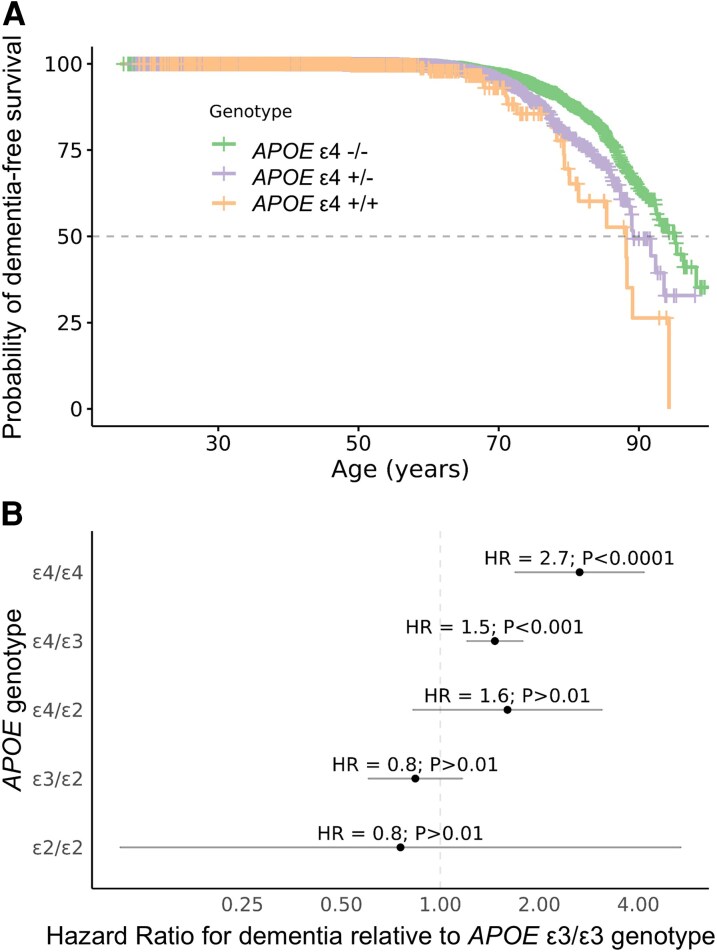
**Association between *APOE* alleles and all-cause dementia in ∼50 000 British south asians**. (A) Kaplan–Meier survival curves indicating the probability of dementia-free survival in carriers of 0, 1, or 2 *APOE* ε4 alleles. (B) Forest plot indicating the Hazard Ratios and 95% confidence intervals for the association of each combination of *APOE* alleles with all-cause dementia, with the ε3/ε3 genotype as the reference. Hazard ratios, confidence intervals and *P* values are derived from a multivariable Cox proportional hazards model estimating the association between *APOE* genotype and the hazard of all-cause dementia. The model was adjusted for age, sex, genetic ancestry and the first ten genetic principal components. Test statistics derived from these models were as follows: ε2/ε2 = −0.28; ε3/ε2 = −1.04; ε4/ε2 = 1.39; ε4/ε3 = 3.82; ε4/ε4 = 4.23.

Carriage of the *APOE* ε4 allele was associated with dementia diagnosis in a dose-dependent fashion (Fig. 2; *APOE* ε4/ε4: HR 2.7, 95% CI 1.7–4.2, *P* < 0.0001; *APOE* ε4/ε3: HR 1.5, 95% CI 1.2–1.8, *P* < 0.001; Cox multivariable regression models adjusted for age at recruitment, gender, PCs 1–10, and genetic ancestry; all models used *APOE* ε3/ε3 as reference).

We observed weaker (i.e. *P* > 0.01) evidence for a risk-increasing association of *APOE* ε4/ε2 (HR 1.6, 95% CI 0.8–3.2, *P* = 0.2) and protective association of the rarer ε2 genotypes *APOE* (ε2/ε2: HR 0.8, 95% CI 0.1–5.4, *P* = 0.8; ε2/ε3: HR 0.8, 95% CI 0.6–1.2, *P* = 0.3). Each copy of the *APOE* ε4 allele was associated with a 55% increase in the hazard of all-cause dementia (HR 1.55, 95% CI 1.33–1.81, *P* = 2.2 × 10^−8^, additive genetic model). In a dominant model, carriage of the *APOE* ε4 allele was associated with a similar degree of risk increase (HR 1.58, 95% CI 1.32–1.90, *P* = 5.6 × 10^−7^).

The magnitude of this association strengthened when considering AD rather than all-cause dementia as the outcome (*APOE* ε4/ε4: HR 6.1, 95% CI 2.4–15.7, *P* = 0.0001; *APOE* ε4/ε3: HR 2.3, 95% CI 1.4–3.7, *P* = 0.001) although these estimates were imprecise owing to the smaller number of cases (N_AD_ = 82; N_Control_ = 51,022). The association between *APOE* status and all-cause dementia was similar in men and women: in gender-stratified models, *APOE* ε4 homozygosity was associated with a substantially elevated risk in both males (HR 2.71, 95% CI 1.54–4.77, *P* = 0.005) and females (HR 2.94, 95% CI 1.37–6.31, *P* = 0.006).

We observed a similar magnitude of association using logistic regression models (ε4/ε4: OR 3.02, 95% CI 1.8–5.1, *P* = 4.9 × 10^−5^; ε4/ε3: OR 1.50, 95% CI 1.2–1.9, *P* = 0.0002). As expected, considering the rare ε2/ε2 genotype as the reference genotype enhanced the effect sizes of genotypes containing ε3 and ε4 alleles, while reducing the precision of the estimates (e.g. ε4/ε4: OR 5.45, 95% CI 0.6–47.0, *P* = 0.12) due to the rarity of this genotype.

To account for bias due to the younger age of the control cohort, we performed a sensitivity analysis restricting to participants recruited over the age of 60 and dementia cases diagnosed >60, yielding a subset of 484 dementia cases (median age at recruitment 74.8, IQR 11.8; median age at diagnostic code report 76.6, IQR 12.7; 39.5% female) and 5015 controls (median age at recruitment 66.0, IQR 9.0; 45.8% female). The association results were similar to the primary analysis (*APOE* ε4/ε4: HR 2.9, 95% CI 1.8–4.8, *P* < 0.0001; *APOE* ε4/ε3: HR 1.6, 95% CI 1.2–1.9, *P* = <0.001; *APOE* ε4/ε2: HR 2.1, 95% CI 1.1- 4.1, *P* = 0.03; *APOE* ε3/ε2: HR 0.9, 95% CI 0.6–1.2, *P* = 0.39; *APOE* ε2/ε2: HR 0.96, 95% CI 0.1–6.8, *P* = 0.97).

### Population attributable fraction

**Figure 3:**
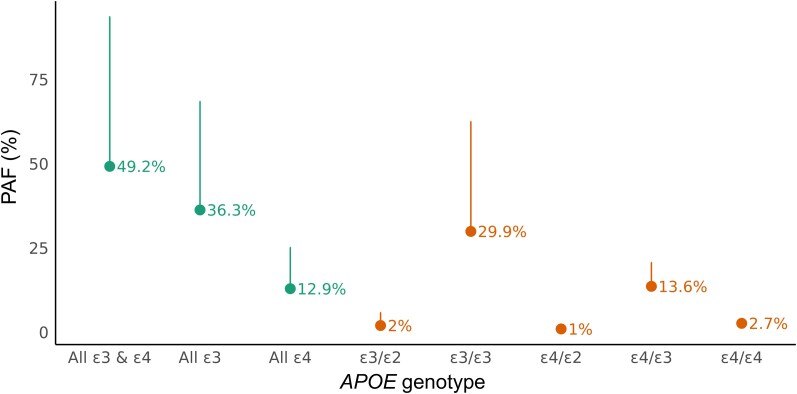
**Population attributable fractions (PAF) for *APOE* alleles.** Points in orange indicate the estimated PAF for each combination of *APOE* genotypes, relative to the low risk ε2/ε2 reference category. Points in dark green indicate the ‘summed’ PAFs, summarising either the contribution of ε4 alleles only, ε3 alleles only, or the joint PAF attributable to both ε4 and ε3. The upper 95% confidence interval is shown. The lower 95% confidence interval is not shown for clarity, as some estimates were imprecise (see text) due to the rarity of the reference genotype and hence large standard errors attached to the estimates.

To quantify the population-level significance of *APOE* variants for dementia in this population, we estimated the PAF for all-cause dementia (Fig. 3). We estimated the PAF for all-cause dementia contingent on the presence of any risk-increasing *APOE* allele (i.e. ε3 or ε4) as 49.2%, however due to the scarcity of ε2/ε2 genotypes the confidence intervals were broad (95% CI −312%–94.0%). We estimated that approximately 12.9% of all-cause dementia cases could be attributed to the presence of *APOE* ε4 (95% CI −5.9%–25.5%) and 36.3% (95% CI −307%–68.4%) to *APOE* ε3.

### Phenome-wide associations of *APOE*

**Figure 4:**
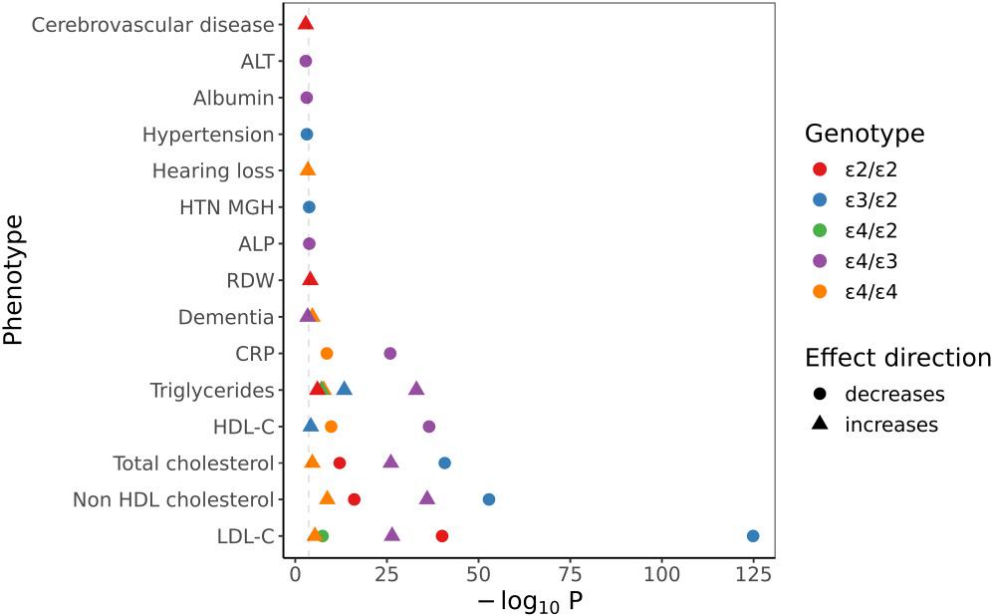
**Phenome-wide associations of *APOE*.** The plot shows the association (from linear or logistic regression models) between each *APOE* genotype and each of the tested quantitative and binary traits in Genes & Health (*n* = 236 traits). Points are shown for genotype-trait associations surpassing a False Discovery Rate of 5%. The dashed line indicates the Bonferroni threshold of 5%. The shape of the point indicates the effect direction, and the x-axis indicates the strength (i.e. -log10[P]). The colour indicates the genotype. All effects are oriented to the reference ε3/ε3 allele. RDW: Red cell distribution width. HTN MGH: Hypertension, alternative codelist definition. ALP: Alkaline phosphatase. ALT: Alanine aminotransferase. CRP: C-reactive protein. HDL-C: High-density lipoprotein cholesterol. LDL-C: Low-density lipoprotein cholesterol.

Dementia-associated *APOE* haplotypes (*APOE* ε4 and ε3) were associated with higher triglycerides, low-density lipoprotein (LDL) cholesterol and total cholesterol, and with lower CRP (Bonferroni-adjusted *P* value < 0.05, total number of traits tested = 236). Other than for these traits and dementia, no other associations persisted at phenome-wide significance (Fig. 4).

## Discussion

Here we demonstrate that the major genetic risk factor for sporadic Alzheimer’s Dementia—*APOE* variation—is associated with all-cause dementia in a biobank-scale cohort of South Asian ancestry, Genes & Health. Specifically, we show that the dementia-associated *APOE* ε4 allele is associated with all-cause dementia in a dose-dependent manner, with homozygotes almost three times as likely to develop dementia as those with the most common ε3/ε3 genotype. The PAF for dementia contingent on the presence of either *APOE* ε3 or ε4**—**the proportion of cases which could theoretically be prevented by neutralising the impact of these variants—was estimated at 42.9% (albeit with wide confidence intervals), analogous to estimates from predominantly White British UK Biobank participants and other predominantly European-ancestry cohorts.^3^

In line with the slightly lower frequency of the ε4 allele in this population than in Europeans, the estimated PAF for ε4 alone is slightly lower than European-ancestry estimates (12.9% in Genes & Health versus 29.3% in UK Biobank—unpublished data, Dylan Williams), although the broad confidence intervals mandate caution in interpreting this difference. Furthermore, we show that the association between *APOE* variants and dementia does not differ substantially by gender, and we replicate the association of *APOE* ε3 and ε4 with elevated triglycerides and LDL cholesterol as expected from previous work.^24^

The major limitations of this work are the relatively young age of the cohort and hence risk of incomplete ascertainment (many participants who will develop dementia in the future have not yet developed it) and the limited phenotyping of dementia cases, which relies on electronic healthcare records and may therefore mix various pathological subtypes of dementia, including misdiagnoses, and does not allow for a detailed examination of dementia phenotypes. These factors may explain why we observe an unexpected male predominance among dementia cases in our cohort.^17^ In addition, as with any volunteer-based study, there are various cultural and other selection biases which might deter people from participating and distort the study cohort with respect to the population: examples of such possible biases include the impact of cultural stigma surrounding cognitive impairment and the impact of subtle cognitive impairment on the ability to take part in the study.

Our use of imputed genetic data rather than sequencing is a limitation, but the very high imputation quality and the fact that both markers used are common are reassuring. Further work with sequencing data will be required to evaluate *APOE* haplotypes beyond the three two-SNP haplotypes examined in this paper, as there may be population-enriched variants seen only in this population which modulate dementia risk in addition to the SNPs encoding the major APOE isoforms.^28,29^

Our findings confirm *APOE* ε4 and ε3 as genetic risk factors for dementia in people of South Asian ancestry and suggest that at the population level, these genetic factors alone account for a sizeable proportion of dementia cases. However, ε4 prevalence, effect size and PAF all showed a trend towards being lower in this British South Asian population than in those of European ancestry, suggesting that *APOE* variation does not fully account for the excess dementia risk that we have previously described in this population.^14^

## Data Availability

All code used to produce these results is available at https://benjacobs123456.github.io/apoe_gh/. Access to Genes & Health data is available on request via https://www.genesandhealth.org/researchers/apply-for-access/.

## Reporting guidelines

This article was prepared in accordance with the STREGA guidelines for genetic association studies.^30^

## Appendix Genes & Health Research Team

Eamonn Maher, Shabana Chaudhary, Joseph Gafton, Karen A Hunt, Shapna Hussain, Kamrul Islam, Mohammed Bodrul Mazid, Elizabeth Owor, Jessry Russell, Nishat Safa, John Solly, Marie Spreckley, David A Van Heel, Jan Whalley, Ishevanhu Zengeya, Emily Mantle, Shaheen Akhtar, Samina Ashraf, Dan Mason, John Wright, Daniel MacArthur, Michael Simpson, Richard C Trembath, Gerome Breen, Raymond Chung, Sang Hyuck Lee, Omar Asgar, Joanne Harvey, Karen Tricker, Caroline Winckley, Hanifa Khatun, Amna Asif, Claudia Langenberg, Grainne Colligan, Ceri Durham, Bill Newman, Ahsan Khan, Hilary Martin, Teng Heng, Matt Hurles, Vivek Iyer, Georgios Kalantzis, Vladimir Ovchinnikov, Iaroslav Popov, Klaudia Walter, Panos Deloukas, David Collier, Ana Angel, Saeed Bidi, Fabiola Eto, Sarah Finer, Chris Griffiths, Sam Hodgson, Benjamin M Jacobs, Rohini Mathur, Caroline Morton, Asma Qureshi, Stuart Rison, Annum Salman, Miriam Samuel, Moneeza K Siddiqui, Daniel Stow, Sabina Yasmin, Julia Zöllner and Sheik Dowlut.
